# Complete Genome Sequence of *Rothia aeria* Type Strain JCM 11412, Isolated from Air in the Russian Space Laboratory Mir

**DOI:** 10.1128/genomeA.01444-16

**Published:** 2016-12-29

**Authors:** Takayuki Nambu, Osamu Tsuzukibashi, Satoshi Uchibori, Kazuyoshi Yamane, Takeshi Yamanaka, Hugo Maruyama, Pao-Li Wang, Naho Mugita, Hiroki Morioka, Kazuya Takahashi, Yutaka Komasa, Chiho Mashimo

**Affiliations:** a Department of Bacteriology, Osaka Dental University, Hirakata, Osaka, Japan; b Department of Microbiology and Immunology, Nihon University School of Dentistry at Matsudo, Chiba, Japan; c Department of Crown Bridge Prosthodontics, Nihon University School of Dentistry at Matsudo, Chiba, Japan; d Department of Geriatric Dentistry, Graduate School of Dentistry, Osaka Dental University, Hirakata, Osaka, Japan; e Department of Geriatric Dentistry, Osaka Dental University, Hirakata, Osaka, Japan

## Abstract

Here, we present the complete genome sequence of* Rothia aeria* type strain JCM 11412, isolated from air in the Russian space laboratory Mir. Recently, there has been an increasing number of reports on infections caused by *R. aeria*. The genomic information will enable researchers to identify the pathogenicity of this organism.

## GENOME ANNOUNCEMENT

Although *Rothia aeria* was first isolated from an air sample in the Russian space laboratory Mir, this organism is mainly found in the oral cavity (1, 2). Even though several case reports have described *R. aeria* as an opportunistic pathogen that causes endocarditis (3–5) and other infections of immunocompromised patients and neonates (6–11), its virulent features remain uncertain. Gram staining to determine the morphological characteristics of *R. aeria* revealed a resemblance to *Nocardia* species (3), which might explain the paucity of isolation from clinical specimens. Molecular approaches are needed to detect and further characterize this organism. Only the 16S rRNA gene-based PCR approach is currently available (12), and neither complete genomic information nor the genetic engineering system is available for this species. Here, we present the full-genome sequence of *R. aeria* type strain JCM 11412.

Total bacterial DNA of strain JCM 11412 was extracted from an overnight culture using a Nucleo spin tissue kit (Macherey-Nagel). A 20-kb SMRTbell library was prepared, and the genome was sequenced using the PacBio RS II system (Pacific Biosciences) on a single-molecule real-time (SMRT) cell using PacBio P6-C4 chemistry.

The *de novo* assembly of 119,399 reads with a mean length of 4,798 bp was completed using the hierarchical genome assembly process (HGAP) algorithm in SMRT Analysis software version 2.3 (13) and revealed two contigs, 1,719,293 bp and 878,081 bp in size with average coverages of 191.68× and 171.9×, respectively. One gap between two contigs was closed by sequencing PCR amplicons from genomic DNA, and the other was manually edited to circularize the overlapping ends of the genome. The final genome sequence is 2,588,680 bp in size and has a G+C content of 56.8%. The genome was then annotated using RAST version 2.0 (14), which successfully identified 2,799 coding sequences, as well as 56 RNA sequences. Of these, 40% of the annotated coding sequences fell within 294 subsystems available in the RAST database. The annotated data set presented here should augment future study of this organism and also provide resources for genetic manipulation.

### Accession number(s).

The genome sequence of *R. aeria* type strain JCM 11412 has been deposited in the DDBJ/EMBL/GenBank database under the accession number AP017895.
